# Supplementary material for: Measuring national capability over big science’s multidisciplinarity: A case study of nuclear fusion research
Source: PLoS One. 2019 Feb 8;14(2):e0211963. doi: 10.1371/journal.pone.0211963 (PMC6368312; doi:10.1371/journal.pone.0211963)
Supplement: S1 Appendix — (PDF) [file pone.0211963.s001.pdf]

S1 Appendix. Determining the number of topics from static LDA model.

The recent work using the regression-based document influence model (rDIM) introduces a method to determine the number of topics K as an input of topic modeling [1]. In general, it runs a static LDA for a large K, and then it specifies the number of significant topics whose corresponding documents have a sufficient number of words larger than w_{th}. In addition to that, we found the minimal K by varying the threshold w_{th}. The details are as follows.

First, we ran a static LDA for $K = 500$ following the reference. In each topic t with $n_d(t)$ corresponding document, we found the number of documents $n_x(t)$ that contained more than w_{th} words (tokens). Then, we determined the significance of the topic from the proportion $p(t) = n_x(t)/n_d(t)$. In the range near the average value of per-document tokens, $p(t)$ has a Gaussian distribution. From the kernel density estimation (KDE) of this Gaussian distribution, we determined the number of significant topics whose proportion of document $p(t)$ is larger than the cutoff proportion, where the derivative of KDE is minimal. By considering the size of tokens in a document, we set the threshold $w_{th} = 50$. As a result, the number of topics was determined as $K = 41$ (S1 Fig).

1. Gerow A, Hu Y, Boyd-Graber J, Blei DM, Evans JA. Measuring discursive influence across scholarship. Proceedings of the National Academy of Sciences. 2018;p. 201719792.
