# Supplementary figures and images for: Measuring national capability over big science’s multidisciplinarity: A case study of nuclear fusion research
Source: PLoS One. 2019 Feb 8;14(2):e0211963. doi: 10.1371/journal.pone.0211963 (PMC6368312; doi:10.1371/journal.pone.0211963)

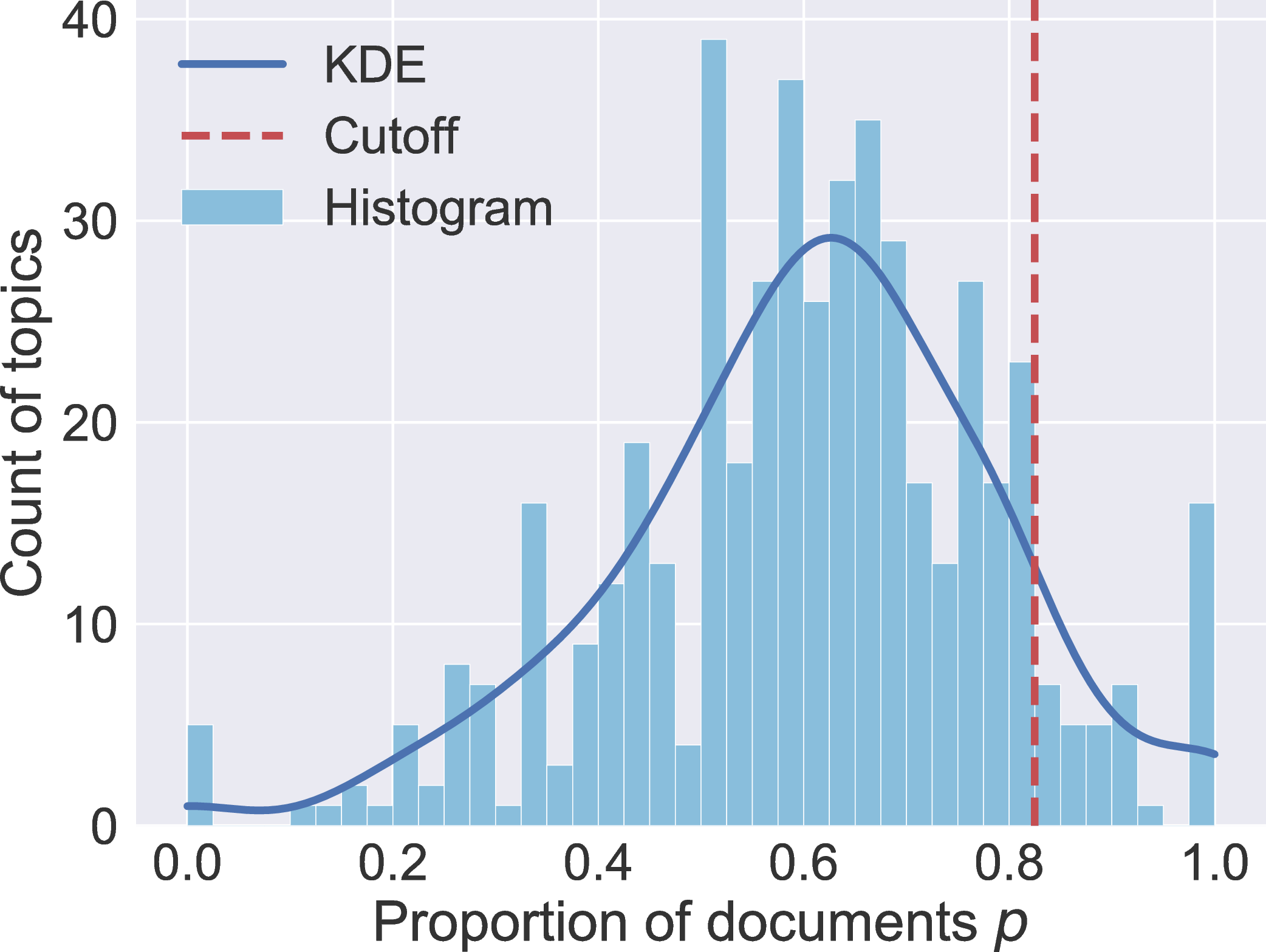

Supplement: S1 Fig — We used the topic usage distribution for static K = 500 model to calculate the cutoff that specifies sufficiently used topics. The minimum of KDE (blue line) derivative determines the cutoff (red dashed line), and the number of topics above this point, K = 41, is used for the DTM. (TIF) [file pone.0211963.s002.tif]
