# Supplementary material for: Measuring national capability over big science’s multidisciplinarity: A case study of nuclear fusion research
Source: PLoS One. 2019 Feb 8;14(2):e0211963. doi: 10.1371/journal.pone.0211963 (PMC6368312; doi:10.1371/journal.pone.0211963)
Supplement: S1 Table — (PDF) [file pone.0211963.s003.pdf]

S1 Table. The top 10 words for 41 topics in nuclear fusion research.

The top 10 words for each topic were obtained from the dynamic topic model on 25,085 abstracts between 1976 and 2016. We manually named 41 topics using the list of the top 15 words of each topic, and the top 10 words among them are listed here.

**Topic**	**Top 10 words**
Wall material, Liquid lithium	wall, material, lithium, tungsten, component, heat, surface, pfc, facing, liquid
Neuturon detector, Spectrum	neutron, detector, jet, measurement, reaction, mev, spectrum, rate, fast, radiation
Neoclassical tearing mode	island, tearing, mhd, instability, neoclassical, surface, mn, magnetohydrodynamic, phase, activity
Transport simulation	model, code, transport, agreement, calculation, numerical, developed, simulated, modelling, compared
Sawtooth crash, Stellarator	sawtooth, crash, helical, stellarator, reconnection, oscillation, lhd, device, configuration, large
Neutral beam injection	beam, neutral, injection, nbi, source, gas, power, injector, efficiency, injected
Resonant magnetic perturbation	perturbation, resonant, rmp, response, pellet, applied, rmps, coil, toroidal, torque
Flux surface, Separatrix	surface, side, region, line, poloidal, inside, separatrix, closed, near, midplane
Plasma flow	flow, toroidal, electric, radial, velocity, poloidal, parallel, zonal, asymmetry, neoclassical
Surface material, Carbon tile	surface, tungsten, sample, layer, carbon, deuterium, hydrogen, retention, material, film
Power, Gyrotron, Tore Supra	power, antenna, system, ghz, rf, mw, gyrotron, tore, supra, transmission
Magnetohydrodynamics	stability, equilibrium, pressure, mhd, ideal, profile, beta, ballooning, kink, toroidal
Internal transport barrier, Steady-state	state, shear, barrier, transport, steady, profile, formation, itb, reversed, bootstrap
Model, Numerical calculation	method, equation, solution, equilibrium, problem, numerical, function, approach, boundary, distribution
Impurity	impurity, runaway, radiation, discharge, gas, disruption, generation, wall, carbon, injection
Diagnostics	system, measurement, diagnostic, resolution, signal, profile, laser, measure, scattering, spatial
Divertor	divertor, heat, target, configuration, power, plate, load, outer, particle, lower
Power supply	power, voltage, supply, system, circuit, loop, arc, breakdown, pulse, kv
Discharge	increase, time, decrease, increasing, increased, value, discharge, observed, change, rate
Lower hybrid current drive	wave, drive, hybrid, lower, lhcd, power, efficiency, frequency, rf, antenna
Soft X-ray, Imaging	xray, camera, reconstruction, measurement, image, imaging, profile, diagnostics, soft, emission
Turbulent transport, Gyrokinetic	transport, profile, gradient, heat, core, particle, turbulent, gyrokinetic, neoclassical, region
Edge-localized mode	elm, hmode, filament, localized, asdex, observed, upgrade, jet, phase, frequency
Alfvén eigenmode, NSTX	spherical, ratio, alfven, aspect, nstx, toroidal, frequency, eigenmodes, torus, gap
Electron cyclotron resonance heating	cyclotron, resonance, ecrh, harmonic, ec, frequency, icrf, power, ech, emission
Geodesic acoustic mode	fluctuation, frequency, gam, amplitude, geodesic, radial, acoustic, structure, observed, correlation
Probe measurement, Scrape-off layer	probe, sol, layer, scrapeoff, measurement, potential, limiter, measured, blob, langmuir
Kinetic theory, Drift	kinetic, rate, linear, drift, growth, gyrokinetic, instability, gradient, regime, model
Spectroscopy	line, emission, spectrum, intensity, spectral, charge, spectroscopy, measurement, nm, measured
Realtime acquisition, EAST	system, data, realtime, east, acquisition, developed, software, signal, operation, time
Application to society (general)	device, role, physic, interaction, process, play, discussed, particular, understanding, application
Feedback control	wall, feedback, coil, system, controller, position, model, vertical, shape, algorithm
Scaling law	parameter, scaling, width, power, value, factor, data, law, model, database
Vacuum vessel, Dust	vacuum, vessel, dust, system, tritium, iter, hydrogen, gas, chamber, safety
Energetic particle loss	particle, loss, fast, energetic, orbit, distribution, fastion, dust, alpha, ripple
Cooling, Magnet	cooling, heat, conductor, magnet, strand, helium, superconducting, test, flow, cable
Disruption	disruption, thermal, force, load, iter, analysis, stress, structure, reactor, method
Power plant, Blanket	reactor, blanket, design, power, system, analysis, tritium, neutron, module, nuclear
ITER, Design, DEMO	iter, design, development, system, reactor, demo, physic, component, project, device
Operation scenario	scenario, operation, power, discharge, drive, performance, iter, profile, limit, regime
Superconducting coil, KSTAR	coil, superconducting, tf, kstar, magnet, system, design, toroidal, vacuum, pf
